# Supplementary material for: Identification and Characterization of Novel Fusion Genes with Potential Clinical Applications in Mexican Children with Acute Lymphoblastic Leukemia
Source: Int J Mol Sci. 2019 May 15;20(10):2394. doi: 10.3390/ijms20102394 (PMC6566803; doi:10.3390/ijms20102394)
Supplement: Supplementary file 1 [file ijms-20-02394-s001.zip › ijms-395346-final SI/Supplementary Figure 1. CREBBP-SRGAP2B fusion transcript.pdf]

CREBBP-SRGAP2B fusion gene

CREBBP 3929832 | *SRGAP2B* 144013900

```
CAAAAGAGCCAAACTCAGCTCGCCCGGTTTCTCGGCGAATGACAGCACAG AGATCCGTGCTCAGCTCACAGAGCAGATGAAATGCCTGGACCAGCAGTGT

CAACCCCAAAAGAGCCAAACTCAGCTCGCCCGGTTTCTCGGCGAATGACAGC
CAACCCCAAAAGAGCCAAACTCAGCTCGCCCGGTTTCTCGGCGAATGACAGCACAG AGATCCGT
CAACCCCAAAAGAGCCAAACTCAGCTCGCCCGGTTTCTCGGCGAATGACAGCACAG AGATCCGT
CAACCCCAAAAGAGCCAAACTCAGCTCGCCCG
CAACCCCAAAAGAGCCAAACTCAGCTCGCCCGGTTTCTCGGCGAATGACAGCACAG AGATCCGTGC
CACCCCCAAAAGAGCCAAACTCAGCTCGCCCGGTTTCTCGGCGAATGACAGCACAG AGCTCCGTGCTCAGCTC
CCAAAAGAGCCAAACTCAGCTCGCCCGGTTTCTCGGCCAATGACAGCAC
AAGAGCCAAACTCAGCTCGCCCGGTTTCTCGGCGAATGACAGCACAG AGCTCCGTGCTCAGCTC
AGAGCCAAACTCAGCTCGCCCGGTTTCTCGGCGAATGACAGCACAG AGATCCGTGCTCAGCTCAC
CAAACTCAGCTCGCCCGGTTTCTCGGCGAATGACAGCACAG AGATCCGTGCTCAGCTCACAGAGCAGATGAAAT
AAACTCAGCTCGCCCGGTTTCTCGGCGAATGACAGCACAG AGATCCGTGCTCAGCTCACAGAGCAGATGTAATG
AACTCAGCTCGCCCGGTTTCTCGGCGAATGACAGCACAG AGATCCGTGCTCAGCTCACAGAGCAGATGAAATG
CAGCTCGCCCGGTTTCTCGGCGAATGACAGCACAG AGATCCGTGCTCAGCTCACAGAGCAGATGAAATGCCTG
CAGCTCGCCCGGTTTCTCGGCGAATGACAGCACAG AGATCCGTGCTCAGCTCACAGAGCAGATGAAATGCCTG
CGGTTTCTCGGCGAATGACAGCACAG AGATCCGTGCTCAGCTCACAGAGCAGATGAAATGCCTGGACCAGC
GGTTTCTCGGCGAATGACAGCACAG AGATCCGTGCTCAGCTCACAGAGCAGATGAAATGCCTGGACCAGCAGTG
GGTTTCTCGGCGAATGACAGCACAG AGATCCGTGCTCAGCTCACAGAGCAGATGAAATGCCTGGACCAGCAGTG
TTCTCGGCGAATGACAGCACAG AGATCCGTGCTCAGCTCACAGAGCAGATG
CGGCGAATGACAGCACAG AGATCCGTGCTCAGCTCACAGAGCAGATGAAATGCCTGGACCAGCAGTGTGAG
CGGCGAATGACAGCACAG AGATCCGTGCTCAGCTCACAGAGCAGATGAAATGCCTGGACCAGCAGTGTGAGCTT
CGGCGAATGACAGCACAG AGATCCGTGCTCAGCTCACAGAGCAGATGAAATGCCTGGACCAGCAGTGTGAGCTT
GCGAATGACAGCACAG AGATCCGTGCTCAGCTCACAGAGCAGATGAAATGCCTGGACCAGCAGTGTGAGCTT
CAGCACAG AGATCCGTGCTCAGCTCACAGAGCAGATGAAATGCCTGGACCAGCAGTGTGAGCTT
CACAG AGATCCGTGCTCAGCTCACAGAGCAGATGAAATGCCTGGCCCAGCAGTGTG
CAGAGATCCGTGCTCAGCTCACAGAGCAGATGAAATGCCTGGACCAGCAGTGTGAGCTT
AGATCCGTGCTCAGCTCACAGAGCAGATGAAATGCCTGGACCAGCAGTGTGAGCTT
```
