# Supplementary material for: Identification and Characterization of Novel Fusion Genes with Potential Clinical Applications in Mexican Children with Acute Lymphoblastic Leukemia
Source: Int J Mol Sci. 2019 May 15;20(10):2394. doi: 10.3390/ijms20102394 (PMC6566803; doi:10.3390/ijms20102394)
Supplement: Supplementary file 1 [file ijms-20-02394-s001.zip › ijms-395346-final SI/Supplementary Figure 2. Continued.pdf]

DNAH14 *225354984* *IKZF1* *50367352*

TTCTGTCTCAATGTAGGGATGCAAGGAATGAAAAATGGAATGGAAGAGAT CCACGACTCTGTCACTCTTGGAGCTTTGCTGTCCTCCCGAGGTGGTGGAG

```
TTCTGTCTCAATGTAGGGATGCAAGGAATGAAAAATGGAATGGAAGAGATC
  CTGTCTCAATGTAGGGATGCAAGGAATGAAAAATGGAATGGAAGAGAT CCACGACTCTGTCACTCTTGGAGCT
      CTCAATGTAGGGATGCAAGGAATGAAAAATGGAATGGAAGAGAT CCACGACTCTGTCACTCTTGGAGCTTTGCT
                                           CCACTTACCCACGACTCTGTCACTCTTGGAGCTTTGCTGTCCTCCCGAGGTGGTGGAGAG
```

DNAH14 *225354984* *IKZF1* *50435762*

TTCTGTCTCAATGTAGGGATGCAAGGAATGAAAAATGGAATGGAAGAGAT GAGAGAAAGATTTGGGAATTATTGCATGGAAATCCCTAAAGTCATCAGCC

```
TGTCTCAATGTAGGGATGCAAGGAATGAAAAATGGAATGGAAGAGATC
    TCAATGTAGGGATGCAAGGAATGAAAAATGGAATGGAAGAGAT GAGAGAAAGATTTGGGAATTATTGCATGG
    TCAATGTAGGGATGCAAGGAATGAAAAATGGAATGGAAGAGAT GAGAGAAAGATTTGGGAATTATTGCATG
              GGATGCAAGGAATGAAAAATGGAATGGAAGAGAT GAGAGAAAGATTTGGGAATTATTGCATGGAAATCCCT
                         AATGAAAAATGGAATGGAAGAGAT GAGAGAAAGATTTGGGAATTATTGCATGGAAATCCCTAAAGTCATCA
                            GAAAAATGGAATGGAAGAGAT GAGAGAAAGATTTGGGAATTATTGCATGGAAATCCCTAAAGTCATCA
                                               TACGAGAGAATGATTTGGGAATTATTGCATGGAAATCCCTAAAGTCATCA
```

DNAH14 *225354984* *IKZF1* *50444490*

TTCTGTCTCAATGTAGGGATGCAAGGAATGAAAAATGGAATGGAAGAGAT CAGTGTGGCTTCTTTTGTGAACCATGAGCACATTGGGCCCGATGCAAATG

```
TTTGTTCTGTCTCAATGTAGGGATGCAAGGAATGAAAAATGGAATGGAAGAGAT CAGTGTGGCTT
TTTGTTCTGTCTCAATGTAGGGATGCAAGGAATGAAAAATGGAATGGAAGAGAT CAGTGTGGCTT
TTTGTTCTGTCTCAATGTAGGGATGCAAGGAATGAAAAATGGAATGGAAGAGATC
TTTGTTCTGTCTCAATGTAGGGATGCAAGGAATGAAAAATGGAATGGAAGAGAT CAGTGTGGCTTCTTTTG
     TCTGTCTCAATGTAGGGATGCAAGGAATGAAAAATGGAATGGAAGAGAT CAGTGTGGCTTCTTTTGTGATCCAT
     TCTGTCTCAATGTAGGGATGCAAGGAATGAAAAATGGAATGGAAGAGAT CAGTGTGGCTTCTTTTGTGAACC
          CTCAATGTAGGGATGCAAGGAATGAAAAATGGAATGGAAGAGAT CAGTGTGGCTTCTTTTGTGAACCATG
          CTCAATGTAGGGATGCAAGGAATGAAAAATGGAATGGAAGAGAT CAGTGTGGCTTCTTTTGTGAACCATG
           TCAATGTAGGGATGCAAGGAATGAAAAATGGAATGGAAGAGAT CAGTGTGGCTTCTTTTGTGAACCATGAGCAC
            CAATGTAGGGATGCAAGGAATGAAAAATGGAATGGAAGAGAT CAGTGTGGCTTCTTTTGTGAACCATGAGC
            CAATGTAGGGATGCAAGGAATGAAAAATGGAATGGAAGAGAT CAGTGTGGCTTCTTTTGTGAACCATGAGCAC
                  AGGGATGCAAGGAATGAAAAATGGAATGGAAGAGAT CAGTGTGGCTTCTTTTGTGAACCATGAGCACATTGGG
                  AGGGATGCAAGGAATGAAAAATGGAATGGAAGAGAT CAGTGTGGCTTCTTTTGTGAACCATGAGCACATTGG
                     GATGCAAGGAATGAAAAATGGAATGGATGAGAT CAGTGTGGCTTCTTTTGTGAACCATGAGCCCATTGGGCCCG
                     GATGCAAGGAATGAAAAATGGAATGGAAGAGAT CAGTGTGGCTTCTTTTGTGAACCATGAGCACATTGGGCCCG
                                 GAAAAATGGAATGGAAGAGAT CAGTGTGGCTTCTTTTGTGAACCATGAGCACATTGGGCCCGATGCAAATGATC
                                    AAATGGAATGGAAGAGAT CAGTGTGGCTTCTTTTGTGAACCATGAGCACATTGGGCCCGATGCAAATGATCCCA
                                    AAATGGAATGGAAGAGAT CAGTGTGGCTTCTTTTGTGAACCATGAGCACATTGGGCCCGATGCAAATGATCCC
                                                GCCTTACCAGTGTGGCTTCTTTTGTGAACCATGAGCACATTGGGCCCGATGCAAATGATCCCA
```

DNAH14 *225374260* *IKZF1* *50448363*

TTTAAGTACAAAAGTAGTACATATAATTGTGAGAAATTTGGAAAATAAAA CCAACTACAGCTCTCATTTGGACAGGGTGGCCTCTCAGGAGAAAGCTCAT

```
AAGTACAAAAGTAGTACATATAATTGTGAGAAATTTGGAAAAT
AAGTACAAAAGTAGTACATATAATTGTGAGAAATTTGGAAAATAAAA CCAACTACAGCTC
      CAAAAGTAGTACATATAATTGTGAGAAATTTGGAAAATAAAA CCAACTACAGCTCTCATTTGGACAGGGTGG
                          TTGTGAGAAATTTGGAAAATAAAA CCAACTACAGCTCTCATTTGGACAGGGTGGCCTCTCAGGAGAAAGCTCAT
                                 GAAATTTGGAAAATAAAA CCAACTACAGCTCTCATTTGGACAGGG
                                 GAAATTTGGAAAATAAAA CCAACTACAGCTCTCATTTGGACAGGGTGGCCTCTCAGG
```
