# Supplementary material for: Identification and Characterization of Novel Fusion Genes with Potential Clinical Applications in Mexican Children with Acute Lymphoblastic Leukemia
Source: Int J Mol Sci. 2019 May 15;20(10):2394. doi: 10.3390/ijms20102394 (PMC6566803; doi:10.3390/ijms20102394)
Supplement: Supplementary file 1 [file ijms-20-02394-s001.zip › ijms-395346-final SI/Supplementary Figure 2. DNAH14-IKZF1 fusion transcript.pdf]

DNAH14-IKZF1 fusion gene

DNAH14 225333860 *IKZF1 50444490*

```
AAACCACTAGTGGGGCCTATGAGGGCTCCACATGAGTGAGGCCAAAAAGC  CAGTGTGGCTTCTTTTGTGAACCATGAGCACATTGGGCCCGATGCAAATG

AGCCTCCTTCCTGGAAACCACTAGTGGGGCCTATGAGGGCTCCACATGAGTGAGGCCAAAAAGCCC
TGCCTCCTTCCTGGAAACCACTAGTGGGGCCTATGAGGGCTCCACATGAGTGAGGCCAAAAAGC CAGTGTGGCT
TGCCTCCTTCCTGGAAACCACTAGTGGGGCCTATGAGGGCTCCACATGAGTGAGGCCAAAAAGC CAGTGTGGC
TGCCTCCTTCCTGGAAACCACTAGTGGGGCCTATGAGGGCTCCACATGAGTGAGGCCAAAAAGC CAGTGTGGCT
    TCCTTCCTGGAAACCACTAGTGGGGCCTATGAGGGCTCCACATGAGTGAGGCCAAAAAGC CAGTGTGGCTTCTT
        TCCTGGAAACCACTAGTGGGGCCTATGAGGGCTCCACATGAGTG
                  CCCTAGGGGGGCCTATGAGGGCTCCACATGAGTGAGGCCAAAAAG
                                          ACATGAGTGAGGCCAAAAAGC CAGTGTGGCTTCTTTTGTGAACCATGAGCACATTGGGCCCGATGCAAATGATC
                                                         GCCTTACCAGTGTGGCTTCTTTTGTGAACCATGAGCACATTGGGCCCGATGCAAATGATCCCACAGATATCAC
```

DNAH14 225333863 *IKZF1 50367352*

```
CCACTAGTGGGGCCTATGAGGGCTCCACATGAGTGAGGCCAAAAAGCCTG  CCACGACTCTGTCACTCTTGGAGCTTTGCTGTCCTCCCGAGGTGGTGGAG

GAAACCACTAGTGGGGCCTATGAGGGCTCCACATGAGTGAGGCCAAAAAGCCTG CCACG
GAAACCACTAGTGGGGCCTATGAGGGCTCCACATGAGTGAGGCCAAAAAGCCTGCTAATTAA
GAAACCACTAGTGGGGCCTATGAGGGCTCCACATGAGTGAGGCCAAAAAGCCTG CCACGACTCTG
GAAACCACTAGTGGGGCCTATGAGGGCTCCACATGAGTGAGGCCAAAAAGCCTG CCACGACTCTGT
GAAACCACTAGTGGGGCCTATGAGGGCTCCACATGAGTG
     CCACTAGTGGGGCCTATGAGGGCTCCACATGAGTGAGGCCAAAAAGCCTG CCACG
      CCCTAGGGGGGCCTATGAGGGCTCCACATGAGTGAGGCCAAAAAG
                GGCCTATGAGGGCTCCACATGAGTGAGGCCAAAAAGCCTG CCACGACTCTGTCACTCTTGGAGCTTTGCTGTCC
                     ATGAGGGCTCCACATGAGTGAGGCCAAAAAGCCTG CCACGACTCTGTCACTCTTGG
                                                   CCACTTACCCACGACTCTGTCACTCTTGGAGCTTTGCTGTCCTCCCGAGGTGGTGGAGAGGTC
                                                      CTTACCCACGACTCTGTCACTCTTGGAGCTTTGCTGTCCTCCCGAGGTGGTGGAGAGGTC
```

DNAH14 225333863 *IKZF1 50444490*

```
CCACTAGTGGGGCCTATGAGGGCTCCACATGAGTGAGGCCAAAAAGCCTG  CAGTGTGGCTTCTTTTGTGAACCATGAGCACATTGGGCCCGATGCAAATG

AAACCACTAGTGGGGCCTATGAGGGCTCCACATGAGTGAGGCCAAAAAGCCTGCTAATTAA
AAACCACTAGTGGGGCCTATGAGGGCTCCACATGAGTGAGGCCAAAAAGCCTG CAGTGTGGCTT
AAACCACTAGTGGGGCCTATGAGGGCTCCACATGAGTGAGGCCAAAAAGCCTG CAGTGTGGCTT
AAACCACTAGTGGGGCCTATGAGGGCTCCACATGAGTGAGGCCAAAAAGCCTG CAGTGTGGCT
AAACCACTAGTGGGGCCTATGAGGGCTCCACATGAGTGAGGCCAAAAAGCCTG CAGTGTGGCT
AAACCACTAGTGGGGCCTATGAGGGCTCCACATGAGTG
AAACCACTAGTGGGGCCTATGAGGGCTCCACATGAGTGAGGCCAAAAAGCCTG CAGTGTGGCTTCTTTTGT
AAACCACTAGTGGGGCCTATGAGGGCTCCACATGAGTGAGGCCAAAAAGCCTG CAGTGTGGCTTCGTTTGT
    CCCTAGGGGGGCCTATGAGGGCTCCACATGAGTGAGGCCAAAAAG
     ACTAGTGGGGCCTATGAGGGCTCCACATGAGTGAGGCCAAAAAGCCTG CAGTGTGGCTTCTTTTGTGAACCATG
        AGTGGGGCCTATGAGGGCTCCACATGAGTGAGGCCAAAAAGCCTG CAGTGTGGCTTCTTTTGTGAACCATGAGC
        AGTGGGGCCTATGAGGGCTCCACATGAGTGAGGCCAAAAAGCCTG CAGTGTGGCTTCTTTTGTGAACCATGAGC
         GTGGGGCCTATGAGGGCTCCACATGAGTGAGGCCAAAAAGCCTG CAGTGTGGCTTCTTTTGTGAACCATGAGCA
           GGGGCCTATGAGGGCTCCACATGAGTGAGGCCAAAAAGCCTG CAGTGTGGCTTCTTTTGTGAACCATGAGCA
             GGCCTATGAGGGCTCCACATGAGTGAGGCCAAAAAGCCTG CAGTGTGGCTTCTTTTGTGAACCATGAGCACAT
                  ATGAGGGCTCCACATGAGTGAGGCCAAAAAGCCTG CAGTGTGGCTTCTTTTGTGAACCATGAGCACATTGGG
                          TCCACATGAGTGAGGCCAAAAAGCCTG CAGTGTGGCTTCTTTTGTGAACCATGAGCACATTGGGCCCGATGC
                                  AGTGAGGCCAAAAAGCCTG CAGTGTGGCTTCTTTTGTGAACCATGAGCACATTGGGCCCGATGCAAATGAT
                                            AAAAGCCTG CAGTGTGGCTTCTTTTGTGAACCATGAGCACATTGGGCCCGATGCAAATGAT
                                               GCCTTACCAGTGTGGCTTCTTTTGTGAACCATGAGCACATTGGGCCCGATGCAAATGAT
                                                      CAGTGTGGCTTCTTTTGTGAACCATGAGCACATTGGGCCCGATGCAAATGAT
```

DNAH14 225346497 *IKZF1 50444230*

```
TACATGACTGATGCTGGCACTGGAACTGCTCACTGCCTCTGCTATTTCTG  CCAGTAATGTTAAAGTAGAGACTCAGAGTGATGAAGAGAATGGGCGTGCC

ATACCAATACATGACTGATGCTGGCACTGGAACTGCTCACTGCCTCTGCTATTTCTGGT
ATACCAATACATGACTGATGCTGGCACTGGAACTG
                           GGAACTGCTCACTGCCTCTGCTATTTCTG CCAGTAATGTTAAAGTAGAGACTCAGAGTGATGAAGAGAATGGG
                                                     TTCAGCCAGTAATGTTAAAGTAGAGACTCAGAGTGATGGAGAGAATGGGCGTGCCTGTGAA
                                                          CCAGTAATGTTAAAGTAGAGACTCAGAGTGATGAAGAGAATGGGCGTGCCTGTGAA
```

DNAH14 225347499 *IKZF1 50367352*

```
CTTTTGAAATGAGGATAATAGTGTCTGTTCTTCCCACTTTGAAAGGGTGC  CCACGACTCTGTCACTCTTGGAGCTTTGCTGTCCTCCCGAGGTGGTGGAG

TTTCATCTTTTGAAATGAGGATAATAGTGTCTGTTCTTCCCACTTTGAAAGGGTGC CCACGACT
            ATGAGGATAATAGTGTCTGTTCTTCCCACTTTGAAAG
                           CTGTTCTTCCCACTTTGAAAGGGTGC CCACGACTCTGTCACTCTTGGAGCTTTGCTGTCCTCCCGAGGTGGTGG
                           CTGTTCTTCCCACTTTGAAAGGGTGC CCACGACTCTGTCACTCTTGGAGCTTTGCTGTCCTCCCGAGGTGGTGG
                               TCTTCCCACTTTGAAAGGGTGC CCACGACTCTGTCACTCTTGGAGCTTTGCTGTCCTCCCGAGGTGGTGG
                               TCTTCCCACTTTGAAAGGGTGC CCACGACTCTGTCACTCTTGGAGCTTTGCTGTCCTCCCGAGGTGGTGG
                                                CCACTTACCCACGACTCTGTCACTCTTGGAGCTTTGCTGTCCTCCCGAGGTGGTGGAGAGGTCCTCG
```
