# Supplementary material for: Identification and Characterization of Novel Fusion Genes with Potential Clinical Applications in Mexican Children with Acute Lymphoblastic Leukemia
Source: Int J Mol Sci. 2019 May 15;20(10):2394. doi: 10.3390/ijms20102394 (PMC6566803; doi:10.3390/ijms20102394)
Supplement: Supplementary file 1 [file ijms-20-02394-s001.zip › ijms-395346-final SI/Supplementary Figure 3. ETV6-SNUPN fusion transcript.pdf]

ETV6-SNUPN fusion gene

ETV6 11905512 | *SNUPN* 75913396

```
    TCGAGCGCTCAGGATGGAGGAAGACTCGATCCGCCTGCCTGCGCACCTGC GGAAGATGGAAGAGTTGAGTCAGGCCCTGGCTAGTAGCTTTTCTGTGTCT

TGCCTCG
TGCCTCGAGCG
TGCCTCGAGCGCTCAGGATGGAGG
TGCCTCGCGCGCTCAGGATCGAGGAAGACTCG
TGCCTCGAGCGCTCAGGATGGAGGAAGACTCGATCCGC
TGCCTCGAGCGCTCAGGATGGAGGAAGACTCGATCCGCCTGCCTGCGC
TGCCTCGAGCGCTCAGGATGGAGGAAGACTCGATCCGCCTGCCTGCGCACCTG
     CGAGCGCTCAGGATGGAGGAAGACTCGATCCGCCTGCCTGCGCACCTGC GGAAGATGGAAGAGTTGAGTCAG
       AGCGCTCAGGATGGAGGAAGACTCGATCCGCCTGCCTGCGCACCTGC GGAAGATGGAAGAGTTGAGTCAGGCCC
                        AAGACTCGATCCGCCTGCCTGCGCACCTGC GGAAGATGGAAGAGTTGAGTCAGGCCCTGGCTAGTAGCTTTTCT
                         AGACTCGATCCGCCTGCCTGCGCACCTGC GGAAGATGGAAGAGTTGAGTCAGGCCCTGGCTAGTAGCTTTTCTG
                              CGATCCGCCTGCCTGCGCACCTGC GGAAGATGGAAGAGTTGAGTCAGGCCCTGGCTAGTAGCTTTTCTGTGTCT
                                                       GGAAGATGGAAGAGTTGAGTCAGGCCCTGGCTAGTAGCTTTTCTGTGTCTCAA
                                                           GATGGAAGAGTTGAGTCAGGCCCTGGCTAGTAGCTTTTCTGTGTCTCAA
                                                               GAAGAGTTGAGTCAGGCCCTGGCTAGTAGCTTTTCTGTGTCTCAA
                                                                     TTGAGTCAGGCCCTGGCTAGTAGCTTTTCTGTGTCTCAA
                                                                           CAGGCCCTGGCTAGTAGCTTTTCTGTGTCTCAA
                                                                                CCTGGCTAGTAGCTTTTCTGTGTCTCAA
                                                                                   GGCTAGTAGCTTTTCTGTGTCTCAA
                                                                                      TAGTAGCTTTTCTGTGTCTCAA
                                                                                         TAGCTTTTCTGTGTCTCAA
                                                                                            CTTTTCTGTGTCTCAA
                                                                                                           A
```
