# Supplementary material for: Identification and Characterization of Novel Fusion Genes with Potential Clinical Applications in Mexican Children with Acute Lymphoblastic Leukemia
Source: Int J Mol Sci. 2019 May 15;20(10):2394. doi: 10.3390/ijms20102394 (PMC6566803; doi:10.3390/ijms20102394)
Supplement: Supplementary file 1 [file ijms-20-02394-s001.zip › ijms-395346-final SI/Supplementary Figure 4. ETV6-NUFIP1 fusion transcript.pdf]

ETV6-NUFIP1 fusion gene

ETV6 11803093	*NUFIP1* 45540070
CTCTCTCGCTGTGAGACATGTCTGAGACTCCTGCTCAGTGTAGCATTAAG	AAACTATCCAACTCTGGCCAATATTGAAAGGAAGAAGAAGTTAAAACTTG
CCTGATCTCTCTCGCTGTGAGACATGTCTGAGACTCCTGCTCAGTGTAGCATT	
CCTGATCTCTCTCGCTGTGAGACATGTCTGAGACTCCTGCTCAGTGTAGCATTAAG	AAACTATCC
CCTGATCTCTCTCGCTGTGAGACATGTCTGAGACTCCTGCTCAGTGTAGCATTAAG	AAACTATCCAACTCTGG
TGATCTCTCTCGCTGTGAGACATGTCTGAGACTCCTGCTCAGTGTAGCATTAAG	AAACTATCCAACTCTGGCC
TGATCTCTCTCGCTGTGAGACATGTCTGAGACTCCTGCTCAGTGTAGCATTAAG	AAACTATCCAACTCTGGCC
ATCTCTCTCGCTGTGAGACATGTCTGAGACTCCTGCTCAGTGTAGCATTAAG	AAACTATCCACCTCTGGCCAAT
TCTCTCTCGCTGTGAGACATGTCTGAGACTCCTGCTCAGTGTAGCATTAAG	AAACTATCCAACTCTGGCCAAT
CGCTGTGAGACATGTCTGAGACTCCTGCTCAGTGTAGCATTAAG	AAACTATCCAACTCTGG
CGCTGTGAGACATGTCTGAGACTCCTGCTCAGTGTAGCATTAAG	AAACTATCCAACTCTGGCC
CTGTGAGACATGTCTGAGACTCCTGCTCAGTGTAGCATTAAG	AAACTATCCAACTCTGGCCAATATTGAAAGG
GTGAGACATGTCTGAGACTCCTGCTCAGTGTAGCATTAAG	AAACTATCCAACTCTGGCCAATATTGAAAGGA
GACATGTCTGAGACTCCTGCTCAGTGTAGCATTAAG	AAACTATCCAACTCTGGCCAATATTGAAAGGA
CCTGCTCAGTGTAGCATTAAG	AAACTATCCAACTCTGGCCAATATTGAAAGGAAGAAGAAGTTAAAACTTGA
GTAGCATTAAG	AAACTATACAACTCTGGCCAATATTGAAAGGAAGAAGAAGTTAAAACTTGAAAAGG
GTAGCATTAAG	AAACTATCCAACTCTGGCCAATATTGAAAGGAAGAAGAAGTTAAAACTTGAAAAGG
GTAGCATTAAG	AAACTATCCAACTCTGGCCAATATTGAAAGGAAGAAGAAGTTAAAACTTGAAAAGG
AGCATTAAG	AAACTATCCAACTCTGGCCAATATTGAAAGGAAGAAGAAGTTAAAACTTGAAAAGG
AGCATTAAG	AAACTATCCAACTCTGGCCAATATTGAAAGGAAGAAGAAGTTAAAACTTGAAAAGG
GCATTAAG	AAACTATCCAACTCTGGCCAATATTGAAAGGAAGAAGAAGTTAAAACTTGAAAAGG
	AACTATCCAACTCTGGCCAATATTGAAAGGAAGAAGAAGTTAAAACTTGAAAAGG
	ATCCAACTCTGGCCAATATTGAAAGGAAGAAGAAGTTAAAACTTGAAAAGG
	CAACTCTGGCCAATATTGAAAGGAAGAAGAAGTTAAAACTTGAAAAGG
