# Supplementary material for: Identification and Characterization of Novel Fusion Genes with Potential Clinical Applications in Mexican Children with Acute Lymphoblastic Leukemia
Source: Int J Mol Sci. 2019 May 15;20(10):2394. doi: 10.3390/ijms20102394 (PMC6566803; doi:10.3390/ijms20102394)
Supplement: Supplementary file 1 [file ijms-20-02394-s001.zip › ijms-395346-final SI/Supplementary Figure 5. EP300-ZNF384 fusion transcript.pdf]

EP300-ZNF385 fusion gene

ZNF384 6788687 | *EP300 41527639*

```
GATAGAAGGCCAGAAGTACGGGTTAGAATTGAAGTGAGATTCTTCCATTC TACTCATGTTGCTCATGGGCCGCATGCCTTGGGGAGACTGCCCAGGCTGC

CCTGAGACTGTGGGATAGAAGGCCAGAAGTACGGGTTAGAATTGAAGTGAGATTCTTCCATT
CCTGAGACTGTGGGATAGAAGGCCAGAAGTACGGGTTAGAATTGAAGTGAGATTCTTCCATTC TACTCATG
CCTGAGACTGTGGGATAGAAGGCCAGAAGTACGGGTTAGAATTGAAGTGAGATTCTTCCATTCTAC
CCTGAGACTGTGGGATAGAAGGCCAGAAGTACGGGTTAGAATTGAAGTGAGATTCTTCCATTC TACTCATGTT
CCTGAGACTGTGGGATAGAAGGCCAGAAGTACGGGTTAGAATTGAAGTGAGATTCTTCCATTC TACTCATGTT
GAGACTGTGGGATAGAAGGCCAGAAGTACGGGTTAGAATTGAAGTGAGATTCTTCCATTC TACTCATGTTGC
GAGACTGTGGGATAGAAGGCCAGCAGTACGGGGTAGAATTGAAGTGAGATTCTTCCATTC TACTCATGTTGC
GGGATAGAAGGCCAGAAGTACGGGTTAGAATTGAAGTGAGATTCTTCCATTC TACTCATGTTGCTCATGGGCCG
GGGATAGAAGGCCAGAAGTACGGGTTAGAATTGAAGTGAGATTCTTCCATTC TACTCATGTTGCTCATGGGCCG
GGGATAGAAGGCCAGAAGTACGGGTTAGAATTGAAGTGAGATTCTTCCATTC TACTCATGTTGCTCATGGGCCG
GGGATAGAAGGCCAGAAGTACGGGTTAGAATTGAAGTGAGATTCTTCCATTC TACTCATGTTGCTCATGGGCCG
GGGATAGAAGGCCAGAAGTACGGGTTAGAATTGAAGTGAGATTCTTCCATTC TACTCATGTTGCTCATGGGCCG
GGATAGAAGGCCAGAAGTACGGGTTAGAATTGAAGTGAGATTCTTCCATTC TACTCATGTTGCTCATGGGCCGC
GGATAGAAGGCCAGAAGTACGGGTTAGAATTGAAGTGAGATTCTTCCATTC TACTCATGTTGCTCATGGGCCGC
GATAGAAGGCCAGAAGTACGGGTTAGAATTGAAGTGAGATTCTTCCATTC TACTCATGTTGCTCATGGGCCGC
GATAGAAGGCCAGAAGTACGGGTTAGAATTGAAGTGAGATTCTTCCATTC TACTCATGTTGCTCATGGGCCGCA
ATAGAAGGCCAGAAGTACGGGTTAGAATTGAAGTGAGATTCTTCCATTC TACTCATGTTGCTCATGGGCCGCAT
AGAAGGCCAGAAGTACGGGTTAGAATTGAAGTGAGATTCTTCCATTC TACTCATGTTGCTCATGGGCCGCATGC
GAAGGCCAGAAGTACGGGTTAGAATTGAAGTGAGATTCTTCCATTC TACTCATGTTGCTCATGGGCCGCATGCC
GGCCAGAAGTACGGGTTAGAATTGAAGTGAGATTCTTCCATTC TACTCATGTTGCTCATGGGCCGCATGCCTTG
GGCCAGAAGTACGGGTTAGAATTGAAGTGAGATTCTTCCATTC TACTCATGTTGCTCATGGGCCGCATGCCTTG
GCCAGAAGTACGGGTTAGAATTGAAGTGAGATTCTTCCATTC TACTCATGTTGCTCATGGGCCGCATGCCTTGG
CAGAAGTACGGGTTAGAATTGAAGTGAGATTCTTCCATTC TACTCATGTTGCTCATGGGCCGCATGCCTTGGGG
CGGGTTAGAATTGAAGTGAGATTCTTCCATTC TACTCATGTTGCTCATGGGCCGCATGCCTTGGGGAGACTGCC
GGTTAGAATTGAAGTGAGATTCTTCCATTC TACTCATGTTGCTCATGGGCCGCATGCCTTGGGGAGACTGCCC
GGTTAGAATTGAAGTGAGATTCTTCCATTC TACTCATGTTGCTCATGGGCCGCATGCCTTGGGGAGACTGCCCA
AGAATTGAAGTGAGATTCTTCCATTC TACTCATGTTGCTCATGGGCCGCATGCCTTGGGGAGACTGCCCAGG
ATAATTGAAGTGAGATTCTTCCATTC TACTCATGTTGCTCATGGGCCGCATGCCTTGGAGAGACTGCCCAGGCT
GAAGTGAGATTCTTCCATTC TACTCATGTTGCTCATGGGCCGCATGCCTTGGGGAGACTGCCCAGGCTGCTGAT
GAAGTGAGATTCTTCCATTC TACTCATGTTGCTCATGGGCCGCATGCCTTGGGGAGACTGCCCAGGCTGCTGAT
GAAGTGAGATTCTTCCATTC TACTCATGTTGCTCATGGGCCGCATGCCTTGGGGAGACTGCCCAGGCTGCTG
GAGATTCTTCCATTC TACTCATGTTGCTCATGGGCCGCATGCCATGGGGAGACTGCCCAGGCTGCTGATTCTGC
GAGATTCTTCCATTC TACTCATGTTGCTCATGGGCCGCATGCCTTGGGGAGACTGCCCAGGCTGCTGATTCTGC
TCCATTC TACTCATGTTGCTCATGGGCCGCATGCCATGGGGAGACTGCCCAGGCTGCTGATTCTGCTGGTTCT
TCCATTC TACTCATGTTGCTCATGGGCCGCATGCCTTGGGGAGACTGCCCAGGCTGCTGATTCTGCTGGTTCT
TCCATTC TACTCATGTTGCTCATGGGCCGCATGCCTTGGGGAGACTGCCCAGGCTGCTGATTCTGCTGGTTCT
TCCATTC TACTCATGTTGCTCATGGGCCGCATGCCTTGGGGAGACTGCCCAGGCTGCTGATTCTGCTGGTTAT
TCCATTC TACTCATGTTGCTCATGGGCCGCATGCCTTGGGGAGACTGCCCAGGCTGCTGATTCTGCTGGTTCT
CCATTC TACTCATGTTGCTCATGGGCCGCATGCCTTGGGGAGACTGCCCAGGCTGCTGATTCTGCTGGTTCT
CCATTC TACTCATGTTGCTCATGGGCCGCATGCCTTGGGGAGACTGCCCAGGCTGCTGATTCTGCTGGTTCT
CCATTC TACTCATGTTGCTCATGGGCCGCATGCCTTGGGGAGACTGCCCAGGCTGCTGATTCTGCTGGTTCT
CATTC TACTCATGTTGCTCATGGGCCGCATGCCTTGGGGAGACTGCCCAGGCTGCTGATTCTGCTGGTTCT
[illegible]
```
