# Supplementary material for: Identification and Characterization of Novel Fusion Genes with Potential Clinical Applications in Mexican Children with Acute Lymphoblastic Leukemia
Source: Int J Mol Sci. 2019 May 15;20(10):2394. doi: 10.3390/ijms20102394 (PMC6566803; doi:10.3390/ijms20102394)
Supplement: Supplementary file 1 [file ijms-20-02394-s001.zip › ijms-395346-final SI/Supplementary Figures_6745.pdf]

International Journal of *Molecular Sciences*

MDPI

Supplementary Figures

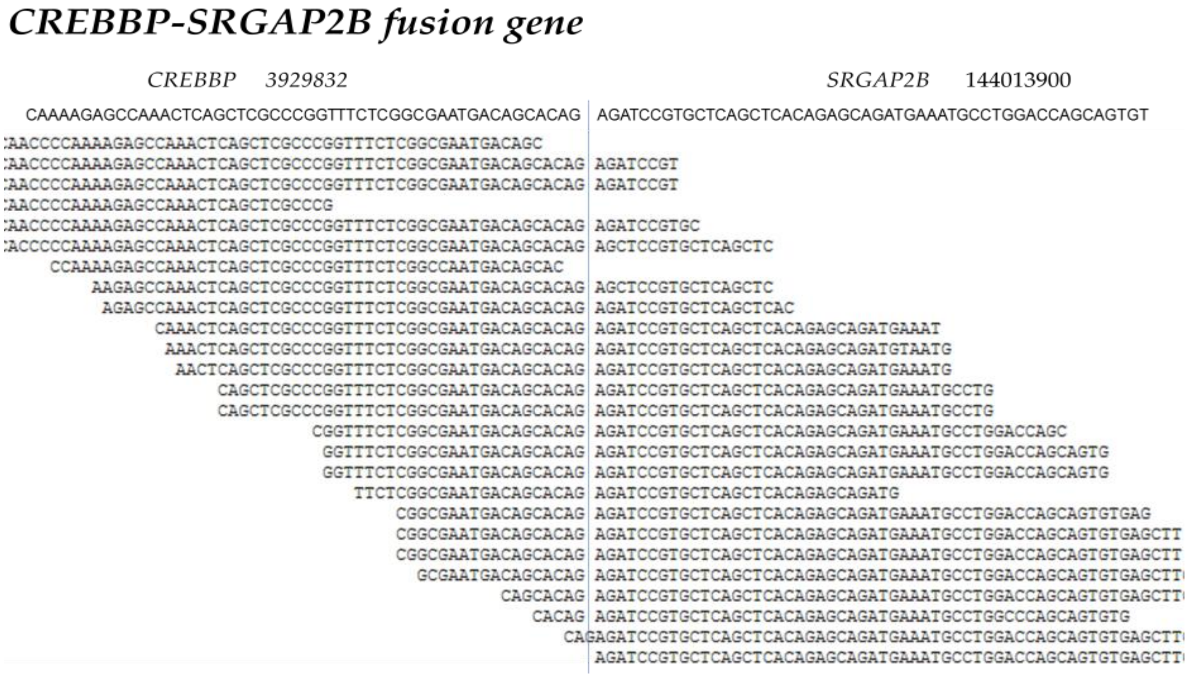

Supplementary Figure 1. ***CREBBP-SRGAP2B*** **fusion transcript.** Alignment of sequence reads mapping across the *CREBBP-SRGAP2B* fusion gene. The nucleotide position and sequence of the assembled fusion transcript appears at the top and reads are showing below it and the vertical line indicates the fusion point. The data were obtained from RNA-seq analysis of the 74MO case transcriptome using the TopHat-Fusion.

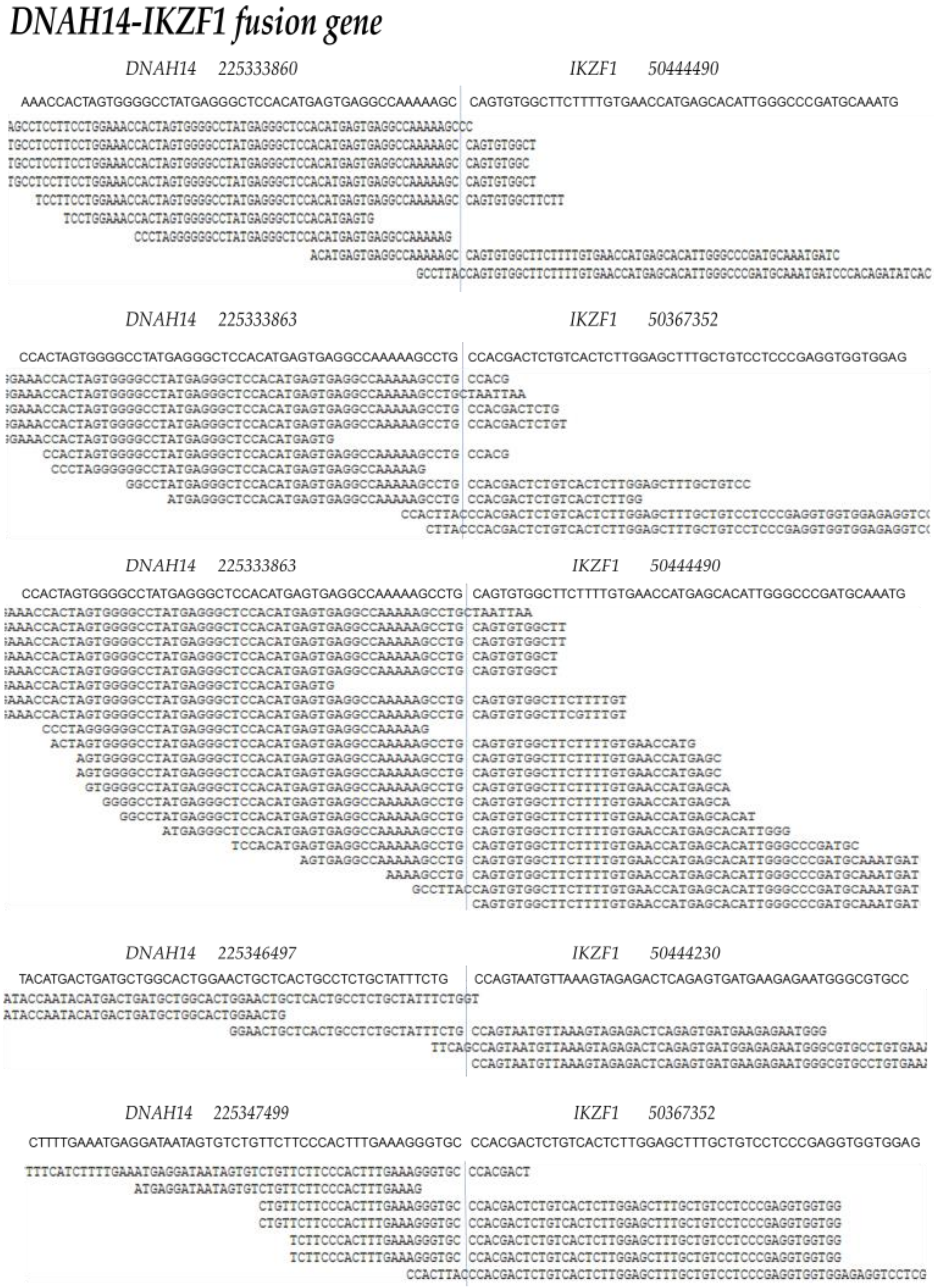

Supplementary Figure 2. ***DNAH14-IKZF1* fusion transcript.** Alignment of sequence reads mapping across the *DNAH14-IKZF1* fusion genes. The nucleotide position and sequence of the assembled fusion transcript appears at the top and reads are showing below it and the vertical line indicates the fusion point. The data were obtained from RNA-seq analysis of the 179MO case transcriptome using the TopHat-Fusion.

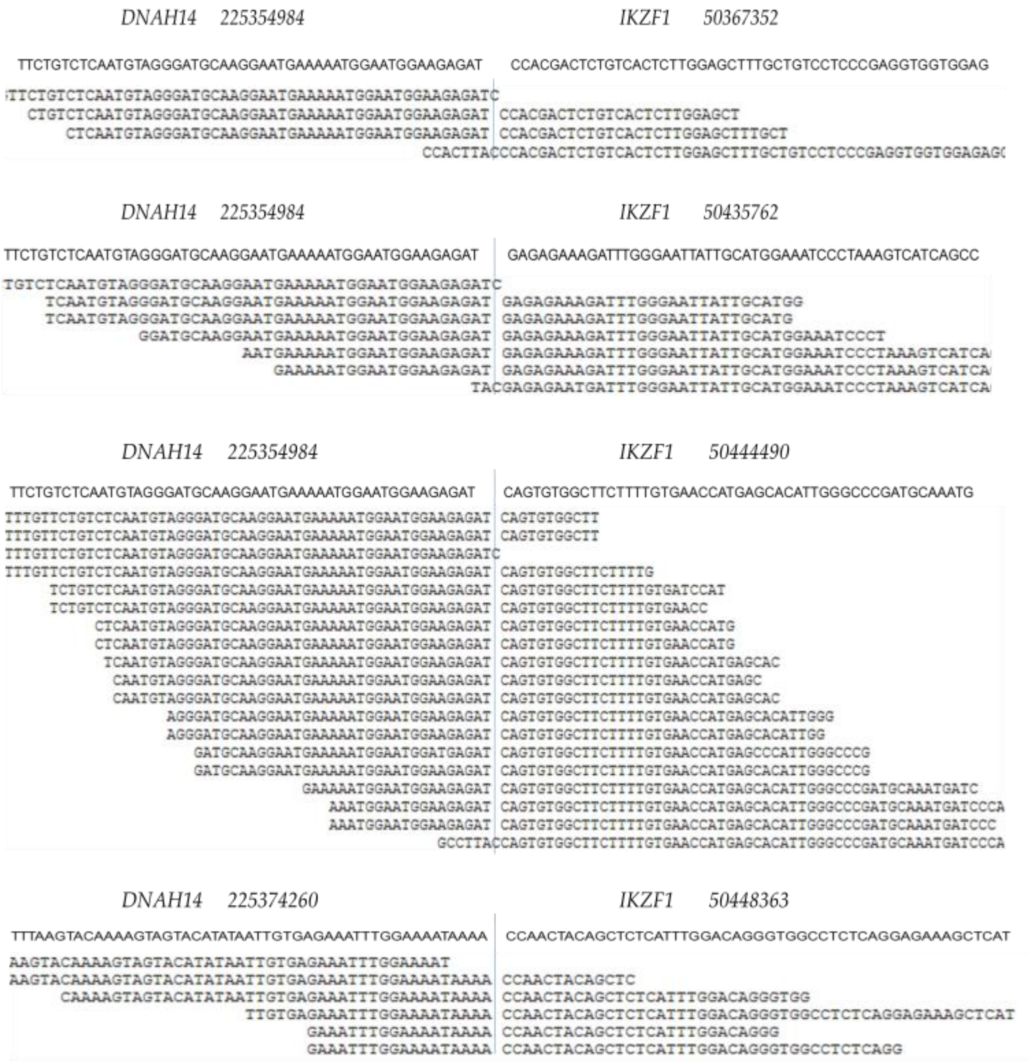

Supplementary Figure 2. Continued

ETV6-SNUPN fusion gene

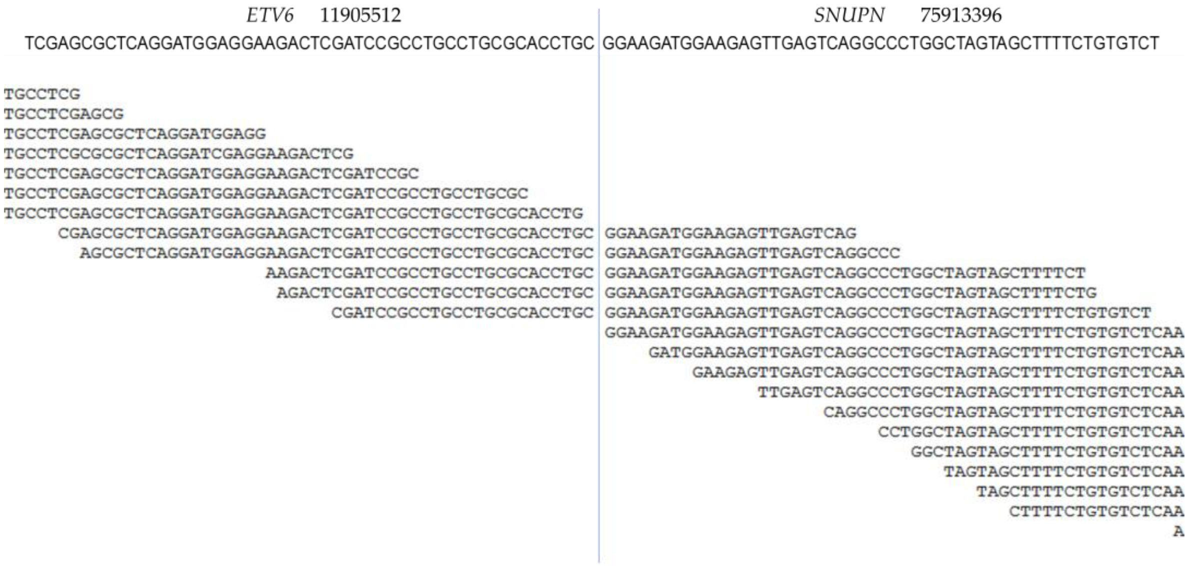

Supplementary Figure 3. ***ETV6-SNUPN*** **fusion transcript.** Alignment of sequence reads mapping across the *ETV6-SNUP* fusion gene. The sequence of the assembled fusion transcript appears at the top and reads are showing below it and the vertical line indicates the fusion point. The data were obtained from RNA-seq analysis of the 28MO case transcriptome using the TopHat-Fusion.

ETV6-NUFIP1 fusion gene

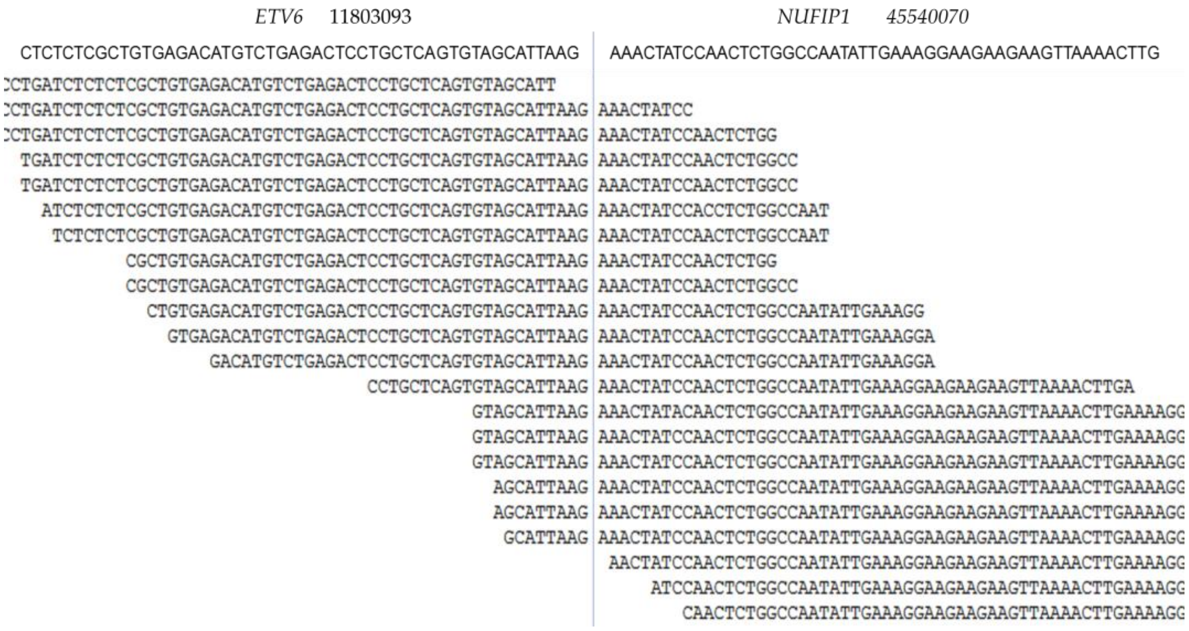

Supplementary Figure 4. ***ETV6-NUFIP1*** **fusion transcript.** Alignment of sequence reads mapping across the *ETV6-NUFIP1* fusion gene. The nucleotide position and sequence of the assembled fusion transcript appears at the top and reads are showing below it and the vertical line indicates the fusion point. The data were obtained from RNA-seq analysis of the 28MO case transcriptome using the TopHat-Fusion.

EP300-ZNF385 fusion gene

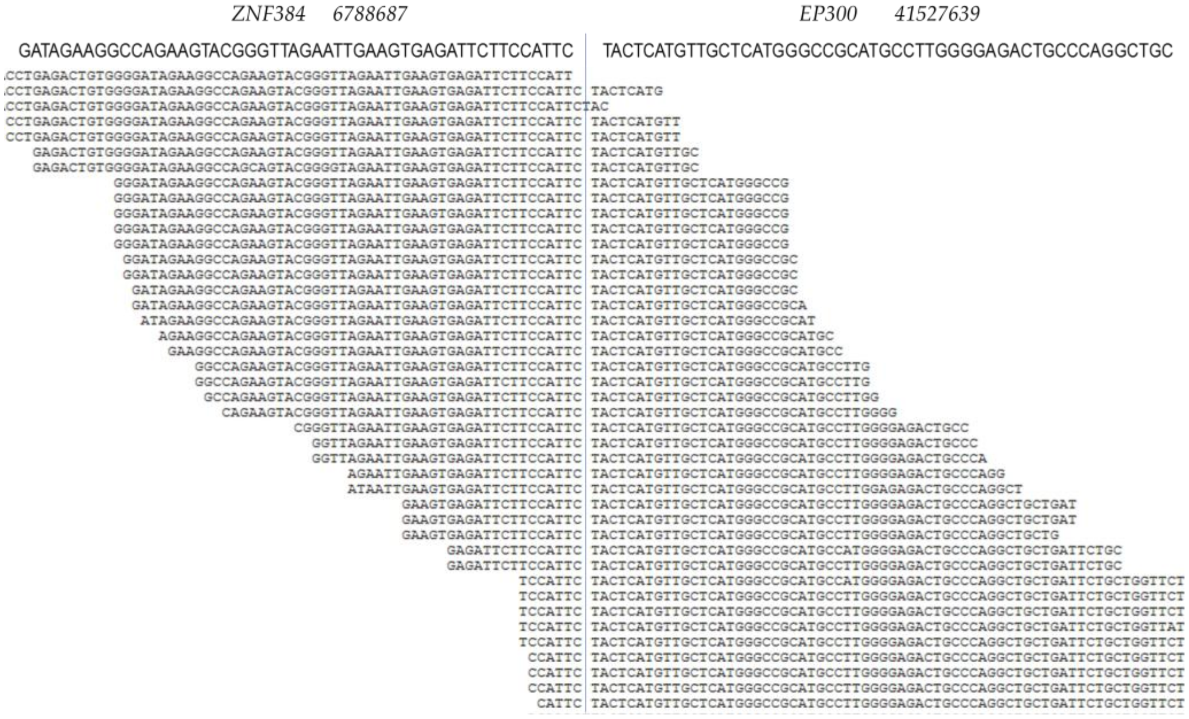

Supplementary Figure 5. *EP300-ZNF384* fusion transcript. Alignment of sequence reads mapping across the *EP300-ZNF384* fusion gene. The nucleotide position and sequence of the assembled fusion transcript appears at the top and reads are showing below it and the vertical line indicates the fusion point. The data were obtained from RNA-seq analysis of the 197MO case transcriptome using the TopHat-Fusion.
