# Supplementary material for: Identification and Characterization of Novel Fusion Genes with Potential Clinical Applications in Mexican Children with Acute Lymphoblastic Leukemia
Source: Int J Mol Sci. 2019 May 15;20(10):2394. doi: 10.3390/ijms20102394 (PMC6566803; doi:10.3390/ijms20102394)
Supplement: Supplementary file 1 [file ijms-20-02394-s001.zip › ijms-395346-final SI/Supplementary materials Table 1. Overview of primer sequences for validation.pdf]

Supplementary materials Table 1. Overview of primer sequences for validation		
Primers (5′-3′)	Sequence	Product size in silico (bp)
ETV6(NUFIP1)_F_int	TCCTGATCTCTCTCGCTGTG	118
NUFIP1 _R_int	TGCTCCTCTCTTCTCCTTTTCA	
ETV6(NUFIP1)_F_ext	GGGAGAGATGCTGGAAGAAA	269
NUFIP1 _R_ext	ATGTCTGGACATCCCCTTCA	
ETV6(SNUPN)_F_int	GCTCAGGATGGAGGAAGACTC	112
SNUPN_R_int	GATCTTGAGACACAGAAAAGCTACT	
ETV6(SNUPN)_F_ext	TCCTGATCTCTCTCGCTGTG	339
SNUPN_R_ext	GCTCACTCTGCTCCAAGGAA	
DNAH14_F	GCAGGGTGCCAAAACACTTT	306
IKZF1_R	CACGGAAGGGGGTCTTTCC	
SRGAP2B F_int	TCGGAAGAAGTCCTGGAGGT	201
CREBBP_R_int	TGTTTTCGCGAGCAGGTGAA	
ZNF384_F_ext	GGCAACAGCTGATCCTTCAT	106
EP300_R_ext	AGAGCCTATGCAGCTCTTGG	
ZNF384_F_int	GGGGATAGAAGGCCAGAAGT	250
EP300_R_int	ATCAGCAGCCTGGGCAGT	
SUDS3P1_F_ext	GCAATCAGGTTCTCTTTCAGC	218
SUDS3_R_ext	TGCAACAACTGCAAGAAGGT	
SUDS3P1_F_int	GCTGGAGGAAGAGTTTCTGC	117
SUDS3_R_int	ACGGAATGCAGAGGGGAATA	
WDR74_F_int	GGAGATGGAATAGGAGCTTGC	101
RCC1_R_int	CTATGCTCCCCTACGCCACT	
WDR74_F_ext	GAGGCGACCGAAGTAAAGGT	271
RCC1_R_ext	ACGTTGTGGAAAGGGACTTG	
GLYR1_F_int	GAGGAAGAGCCAGTCTGTCG	105
SLC9A8_R_int	CCTTGGACTATGTTTACAATCAGC	
GLYR1_F_ext	GGCCACATCTCCTCTGTTTC	264
SLC9A8_R_ext	AGGGTCAGCACCTCAGTGAT	
